# Arthroscopic and MRI Visibility of the Rotator Cable in Supraspinatus Tears: Agreement, Associated Factors, and Relationship with Preoperative Range of Motion

**DOI:** 10.3390/jcm15176535

**Published:** 2026-08-24

**Authors:** Zübeyir Akkoyun, Cüneyd Günay, Mahircan Demir, Cüneyt Çalışır, Ertuğrul Çolak

**Affiliations:** 1 Department of Orthopedics and Traumatology, Faculty of Medicine, Eskişehir Osmangazi University, Eskisehir 26480, Türkiye; 2 Department of Orthopedics and Traumatology, Mamak State Hospital, Ankara 06270, Türkiye; 3 Department of Radiology, Faculty of Medicine, Eskişehir Osmangazi University, Eskisehir 26480, Türkiye; 4 Department of Biostatistics, Faculty of Medicine, Eskişehir Osmangazi University, Eskisehir 26480, Türkiye

**Keywords:** rotator cable, rotator cuff tear, arthroscopy, magnetic resonance imaging, supraspinatus, shoulder, range of motion, suspension bridge model

## Abstract

**Background:** The rotator cable is thought to contribute to load transmission and preservation of shoulder function in rotator cuff tears; however, its detectability on arthroscopy and magnetic resonance imaging (MRI), agreement between these modalities, and clinical relevance remain incompletely defined. This study evaluated rotator cable visibility on arthroscopy and MRI, factors associated with arthroscopic cable visibility, and its relationship with preoperative active shoulder motion. **Methods:** This retrospective cross-sectional study included 128 patients who underwent shoulder arthroscopy for supraspinatus tears between January 2019 and February 2023. Arthroscopic video recordings were reviewed for rotator cable visibility. Standardized preoperative MRI review was available in 58 patients. Agreement between MRI and arthroscopic visualization was assessed using Cohen’s kappa and percentage agreement measures. Multivariable binary logistic regression was performed to identify factors independently associated with arthroscopic cable non-visibility. **Results:** The rotator cable was visible arthroscopically in 79 of 128 patients (61.7%) and on MRI in 38 of 58 patients (65.5%). Overall agreement between MRI and arthroscopy was 67.2% (95% CI, 53.7–79.0%), with a Cohen’s kappa of 0.315 (95% CI, 0.090–0.540; *p* = 0.011), indicating fair agreement. Positive and negative percent agreement were 78.8% and 52.0%, respectively. Although increasing age was associated with cable non-visibility in univariable analysis, this association did not remain statistically significant after multivariable adjustment. Higher Lafosse grade was independently associated with cable non-visibility in the overall cohort (adjusted OR, 1.43 per grade; 95% CI, 1.04–1.97; *p* = 0.028), whereas increasing tear size was independently associated with cable non-visibility among patients with full-thickness tears (adjusted OR, 2.78 per category; 95% CI, 1.19–6.52; *p* = 0.019). The unadjusted association between massive tear size and reduced MRI cable visibility did not remain significant after false discovery rate adjustment (q = 0.276). No statistically significant associations were detected between cable visibility or MRI-measured cable dimensions and preoperative active abduction or forward elevation. **Conclusions:** MRI and arthroscopy demonstrated fair agreement in the assessment of rotator cable visibility. After multivariable adjustment, cable non-visibility was more closely associated with tear-related characteristics than with patient age. No statistically significant associations were detected between cable characteristics and the assessed preoperative range-of-motion measures; however, smaller or moderate associations cannot be excluded, particularly within the MRI subgroup. Rotator cable visibility should primarily be interpreted as a marker of detectability and tear morphology rather than as a direct surrogate for structural integrity or shoulder function.

## 1. Introduction

The rotator cable is a ligamentous structure that encircles the rotator crescent at the insertional region of the rotator cuff. It was first described as a “transverse band” by Clark et al. and was later termed the “rotator cable” by Burkhart, who proposed the suspension bridge model to explain its biomechanical role in rotator cuff tears [1,2]. According to this model, the rotator cable may redistribute tensile loads away from the rotator crescent, thereby contributing to preservation of shoulder function even in the presence of substantial rotator cuff defects [1,3,4].

Although the rotator cable has been consistently demonstrated in cadaveric and anatomical studies [5,6], its visualization in clinical practice remains variable. Reported detection rates range from 44% to 100% on magnetic resonance imaging (MRI), 11% to 99% on ultrasonography, and 10% to 100% during arthroscopy [7,8,9]. This wide variability raises questions regarding the reliability of current assessment methods and the factors that influence cable detectability. Accurate identification of the rotator cable may be clinically relevant, as previous studies have suggested that cable integrity may contribute to maintenance of active elevation in massive rotator cuff tears [10,11,12]. Denard et al. reported that disruption of the anterior or posterior cable attachment was associated with pseudoparalysis in patients with massive tears [11]. In addition, biomechanical evidence suggests that suture positioning relative to the rotator cable may influence fixation strength, indicating that intraoperative recognition of the cable may have implications for surgical strategy [13,14].

Despite these potential clinical implications, several aspects of rotator cable assessment remain unclear. Data comparing arthroscopic and MRI visualization of the rotator cable in the same patient cohort are limited [8,9], and the tear-related factors influencing cable visibility on each modality have not been fully characterized. Furthermore, although the rotator cable has been proposed to contribute to shoulder function, the relationship between cable visibility or MRI-measured cable dimensions and preoperative physical examination findings, particularly active range of motion, remains insufficiently defined [11,12].

The purpose of this study was to evaluate the visibility of the rotator cable on arthroscopy and MRI in patients undergoing shoulder arthroscopy for supraspinatus tears, to assess agreement between the two modalities, to identify factors associated with cable visibility, and to determine whether cable visibility or MRI-measured dimensions are associated with preoperative active shoulder range of motion.

## 2. Materials and Methods

### 2.1. Study Design and Ethical Approval

This retrospective cross-sectional observational study was conducted at a tertiary referral center. Ethical approval was obtained from the institutional non-interventional research ethics committee (Decision No. 18; 21 March 2023). The requirement for informed consent was waived by the institutional non-interventional research ethics committee because of the retrospective nature of the study and the use of previously collected clinical, imaging, and intraoperative video data.

### 2.2. Patient Selection

Patients who underwent shoulder arthroscopy between January 2019 and February 2023 were screened for eligibility. All surgical procedures and preoperative physical examinations were performed by a single experienced shoulder surgeon.

The inclusion criteria were:

Age ≥ 18 years;

Intraoperative confirmation of a supraspinatus tendon tear; availability of adequate intraoperative arthroscopic video recordings for assessment of the rotator cable; and availability of preoperative physical examination records including active shoulder abduction and forward elevation.

The exclusion criteria were:

1-Shoulder instability as the primary surgical indication;

2-Absence of a supraspinatus tear on intraoperative assessment;

3-Rotator cuff arthropathy;

4-Revision shoulder surgery;

5-Inadequate arthroscopic visualization or incomplete clinical records.

**Figure 1:**
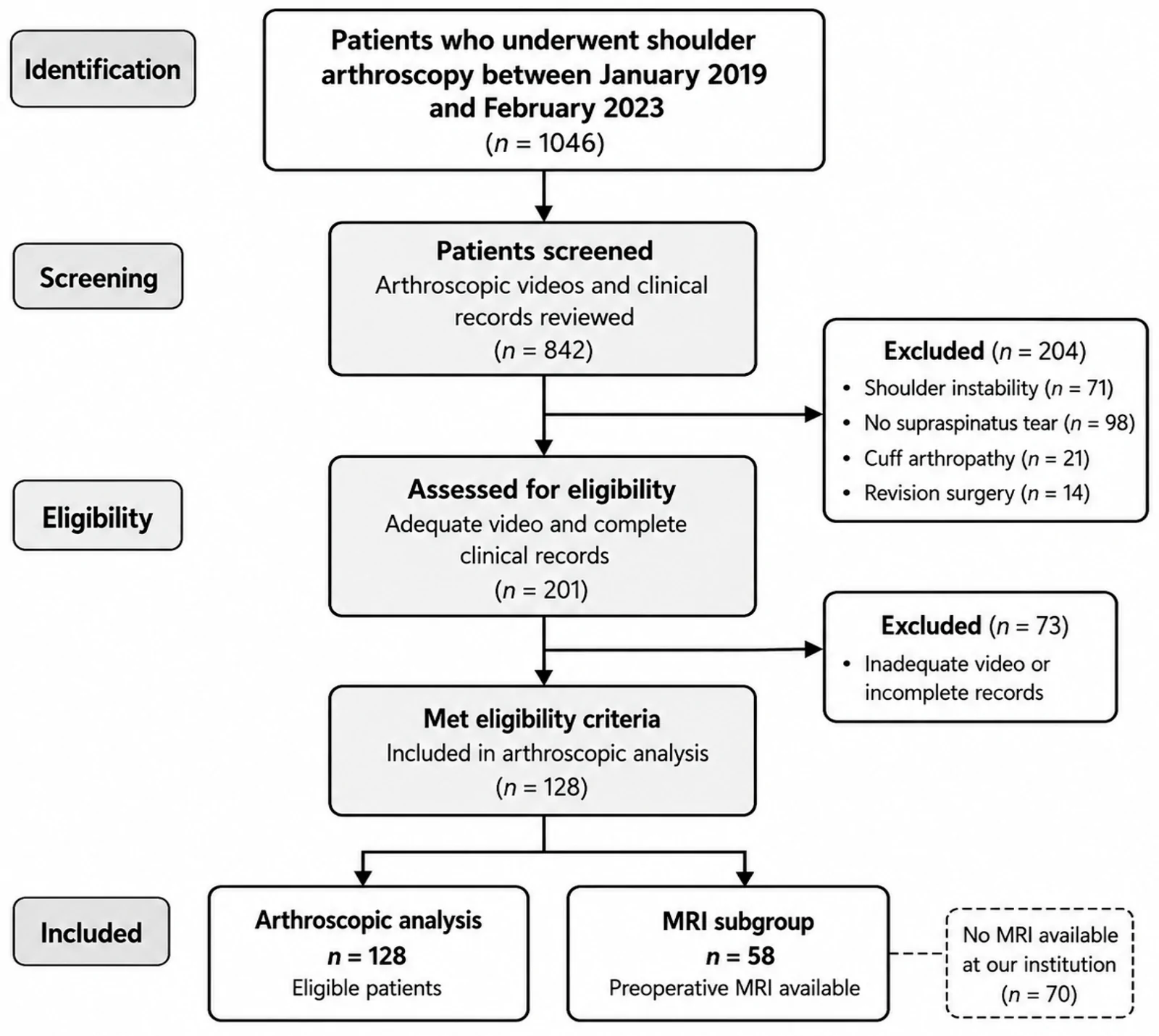
Patient selection flow diagram.

A total of 128 patients met the eligibility criteria and were included in the arthroscopic and clinical analyses. Among these patients, 58 had preoperative MRI studies available for standardized review and were included in the MRI-based analyses. Only preoperative MRI examinations performed at our institution and available in the institutional imaging archive were included in the standardized MRI review. This restriction was applied to minimize heterogeneity in imaging acquisition and to ensure consistent assessment conditions across the MRI subgroup. The patient selection process is summarized in Figure 1.

### 2.3. Arthroscopic Assessment

All procedures were performed with the patient in the beach-chair position using a 30° arthroscope (KARL STORZ SE & Co. KG, Tuttlingen, Germany). The glenohumeral joint and the articular surface of the posterosuperior rotator cuff were systematically evaluated during diagnostic arthroscopy.

Intraoperative arthroscopic video recordings were retrospectively reviewed for rotator cable visibility. The arthroscopic video recordings were evaluated by a single observer who was blinded to the MRI findings and relevant clinical information at the time of assessment. The rotator cable was considered visible when a crescentic, raised fibrous structure was identifiable on the articular side of the posterosuperior rotator cuff, adjacent to the rotator crescent. Cable visibility was recorded as a binary variable: visible or not visible. “Visible” indicated that the predefined morphological structure could be identified on arthroscopic review. “Not visible” indicated failure to identify the structure under the available viewing conditions and was not assumed to represent true anatomical absence, complete structural disruption, or loss of biomechanical competence.

Supraspinatus tears were classified as partial-thickness or full-thickness tears. Full-thickness tears were further classified according to their size using the DeOrio and Cofield classification: small (<1 cm), medium (1–3 cm), large (3–5 cm), and massive (>5 cm) [15]. The anterior extent of full-thickness tears was categorized according to their relationship with the long head of the biceps tendon (LHBT): not reaching the LHBT, reaching the posterior aspect of the LHBT, or extending anterior to the LHBT. Subscapularis tears were graded according to the Lafosse classification [16].

### 2.4. MRI Assessment

Preoperative MRI examinations were performed using a 3-Tesla scanner (GE Discovery 750 W; GE Healthcare, Chicago, IL, USA) with a 64-channel coil. The imaging protocol included oblique coronal fat-suppressed T2-weighted turbo spin-echo and proton density sequences, with a slice thickness of 4 mm, matrix size of 512 × 512, and field of view of 180 mm.

All MRI examinations were reviewed by a musculoskeletal radiologist who was blinded to the arthroscopic findings and clinical information at the time of assessment. The rotator cable was defined as a continuous hypointense band along the articular surface of the supraspinatus and infraspinatus tendons, in continuity with the coracohumeral ligament. MRI visibility was confirmed only when the structure was identifiable on both oblique coronal and oblique sagittal images. MRI non-visibility indicated that the cable could not be confidently identified according to the predefined imaging criteria and did not necessarily indicate anatomical absence or complete structural disruption.

When the rotator cable was visible, its width and thickness were measured in millimeters at the midportion of the cable, primarily on the oblique coronal plane. Width was defined as the mediolateral dimension, and thickness was defined as the craniocaudal dimension.

### 2.5. Physical Examination

Preoperative physical examination records were reviewed for active shoulder range of motion. Active abduction and active forward elevation were recorded in degrees and used as the primary clinical variables for range-of-motion analysis.

### 2.6. Statistical Analysis

Statistical analyses were performed using IBM SPSS Statistics, version 27.0 (IBM Corp., Armonk, NY, USA). Continuous variables were assessed for normality using the Shapiro–Wilk test and visual inspection of histograms and Q–Q plots. Normally distributed variables are presented as mean ± standard deviation, whereas non-normally distributed variables are presented as median and interquartile range. Categorical variables are presented as frequencies and percentages.

Comparisons between two independent groups were performed using the Student *t* test or Mann–Whitney U test, as appropriate. Comparisons among three or more groups were performed using one-way analysis of variance or the Kruskal–Wallis test, depending on data distribution. Categorical variables were compared using the Pearson chi-square test, Yates-corrected chi-square test, or Monte Carlo exact test, as appropriate according to expected cell counts.

To assess potential selection bias related to the availability of MRI examinations, patients included in the standardized MRI analysis were compared with those not included. Continuous variables were compared using the independent-samples *t* test or Mann–Whitney U test, as appropriate. Categorical variables were compared using the Pearson chi-square test or Monte Carlo exact test according to expected cell frequencies. Tear size and tear extension relative to the LHBT were compared only among patients with full-thickness tears, because these variables were not applicable to partial-thickness tears.

Agreement between MRI and arthroscopic visualization of the rotator cable was evaluated using cross-classification and Cohen’s kappa coefficient. Because neither modality represented a true gold standard for structural cable integrity, arthroscopic visualization was used only as an operational comparator. Positive percent agreement, negative percent agreement, and overall percent agreement were calculated with exact binomial 95% confidence intervals. Positive percent agreement was defined as the proportion of arthroscopically visible cables that were also visible on MRI, whereas negative percent agreement was defined as the proportion of cables not visible on arthroscopy that were also not visible on MRI.

Multivariable binary logistic regression analyses were performed to identify factors independently associated with absence of arthroscopic rotator cable visibility. Arthroscopic cable non-visibility was coded as the outcome event (0 = visible; 1 = not visible); therefore, odds ratios greater than 1 indicate increased odds of the cable not being visualized. Because tear size and tear extension relative to the LHBT were defined only for full-thickness tears, two complementary models were constructed. The overall-cohort model included age, sex, tear type (partial- versus full-thickness), and Lafosse grade. The full-thickness-tear model included age, sex, tear size, Lafosse grade, and tear extension relative to the LHBT. Age was expressed per 10-year increase. Tear size was entered as an ordinal variable ranging from 1 (small) to 4 (massive), and Lafosse grade was entered as an ordinal variable ranging from 0 (no subscapularis tear) to 5 (Lafosse Type V). All variables were entered simultaneously using the enter method. Adjusted odds ratios (aORs) with 95% confidence intervals were reported.

Because multiple univariable comparisons were performed when examining factors associated with cable visibility, the Benjamini–Hochberg false discovery rate procedure was applied separately to two predefined families of tests: factors associated with arthroscopic cable visibility and factors associated with MRI cable visibility. Adjusted q values were used to assess the robustness of these univariable findings, whereas the multivariable regression analyses were interpreted separately as adjusted models.

Correlations between MRI-measured cable dimensions and preoperative range of motion were assessed using Spearman’s rank correlation coefficient.

No a priori sample size calculation was performed. Because the study was retrospective, the sample size was determined by the number of eligible patients available during the study period. A sensitivity analysis was conducted using a two-sided alpha level of 0.05 and 80% power to estimate the minimum detectable effect sizes for selected analyses. Minimum detectable standardized mean differences were estimated under a two-sided independent two-group comparison framework, incorporating the observed unequal allocation ratios. The minimum detectable correlation was estimated using a two-sided test of a Pearson correlation coefficient. Based on the available group sizes, the minimum detectable standardized mean difference was approximately 0.51 for comparisons according to arthroscopic cable visibility and 0.79 for comparisons according to MRI cable visibility. For correlation analyses involving 38 patients with MRI measurements, the minimum detectable correlation was approximately |r| = 0.44.

A *p* value of <0.05 was considered statistically significant.

## 3. Results

### 3.1. Patient Demographics and Tear Characteristics

A total of 128 patients were included in the study, including 82 female patients (64.1%) and 46 male patients (35.9%). The mean age was 57.1 ± 10.0 years (range, 32–83 years).

Supraspinatus tears were partial-thickness in 33 patients (25.8%) and full-thickness in 95 patients (74.2%). Among patients with full-thickness tears, 11 tears (11.6%) were small, 36 (37.9%) were medium, 34 (35.8%) were large, and 14 (14.7%) were massive. With regard to the anterior extent of full-thickness tears relative to the long head of the biceps tendon (LHBT), 28 tears (29.5%) did not reach the LHBT, 31 (32.6%) reached the posterior aspect of the LHBT, and 36 (37.9%) extended anterior to the LHBT.

Subscapularis tears were identified in 79 patients (61.7%). According to the Lafosse classification, subscapularis tears were Type I in 7 patients (5.5%), Type II in 33 (25.8%), Type III in 31 (24.2%), Type IV in 6 (4.7%), and Type V in 2 (1.6%).

### 3.2. Comparison of Patients Included and Not Included in the MRI Analysis

Of the 128 patients, 58 were included in the standardized MRI analysis and 70 were not included. No statistically significant differences were detected between the groups in age, sex, arthroscopic cable visibility, active abduction, or active forward elevation (all *p* > 0.05). Partial-thickness tears were more frequent in the MRI subgroup than in the group without standardized MRI review (36.2% vs. 17.1%; *p* = 0.014). Among patients with full-thickness tears, neither the distribution of tear size nor tear extension relative to the LHBT differed significantly between the groups (*p* > 0.05). The comparison is presented in Supplementary Table S1.

### 3.3. Factors Associated with Arthroscopic Cable Visibility

Increasing age was significantly associated with reduced arthroscopic cable visibility (*p* = 0.005). Patients in whom the cable was visible were younger than those in whom it was not visible (median age, 55.0 years; IQR, 49.5–60.5 years vs. 58.0 years; IQR, 54.0–66.0 years). Factors associated with arthroscopic cable visibility are summarized in Table 1.

Advancing subscapularis tear grade was significantly associated with reduced arthroscopic cable visibility (*p* = 0.011). Cable visibility rates were 79.6% in patients without subscapularis tears, 57.1% in Lafosse Type I tears, 60.6% in Type II tears, 41.9% in Type III tears, 33.3% in Type IV tears, and 50.0% in Type V tears.

Increasing full-thickness tear size was significantly associated with reduced cable visibility (*p* < 0.001). The cable was visible in 90.9% of small tears, 69.4% of medium tears, and 50.0% of large tears. The cable was not identified in any patient with a massive tear.

Partial-thickness tears demonstrated significantly higher cable visibility than full-thickness tears (81.8% vs. 54.7%; *p* = 0.011).

Tear location relative to the LHBT was also significantly associated with arthroscopic cable visibility (*p* < 0.001). Tears extending anterior to the LHBT had a lower cable visibility rate (25.0%) than tears not reaching the LHBT (71.4%), tears reaching the posterior aspect of the LHBT (74.2%), and partial-thickness tears (81.8%). No significant difference in cable visibility was observed among the latter three groups. When tears reaching or extending beyond the LHBT were pooled, cable visibility was lower than in LHBT-sparing tears, but this difference did not reach statistical significance (47.8% vs. 71.4%; *p* = 0.059).

Sex was not significantly associated with arthroscopic cable visibility (*p* = 0.424). After false discovery rate adjustment, the univariable associations of arthroscopic cable visibility with age, Lafosse grade, tear size, tear type, and tear localization relative to the LHBT remained statistically significant. FDR-adjusted q values are provided in Supplementary Table S2.

### 3.4. Multivariable Factors Associated with Arthroscopic Cable Visibility

Multivariable logistic regression results are presented in Table 2. In the overall cohort, increasing Lafosse grade was independently associated with absence of arthroscopic cable visibility (aOR per one-grade increase, 1.43; 95% CI, 1.04–1.97; *p* = 0.028). Age, sex, and full-thickness tear status were not independently associated with cable non-visibility (all *p* > 0.05). The overall model was statistically significant (likelihood-ratio χ^2^(4) = 18.46; *p* = 0.001).

In the analysis restricted to patients with full-thickness tears, increasing tear size was independently associated with absence of arthroscopic cable visibility (aOR per one-category increase, 2.78; 95% CI, 1.19–6.52; *p* = 0.019). Age, sex, Lafosse grade, and tear extension relative to the LHBT were not independently associated with cable non-visibility after adjustment (all *p* > 0.05). The overall model was statistically significant (likelihood-ratio χ^2^(6) = 33.56; *p* < 0.001).

### 3.5. Factors Associated with MRI Cable Visibility

Among the 58 patients with available MRI studies, massive tears showed lower MRI cable visibility than non-massive tears (25.0% vs. 67.9%; unadjusted *p* = 0.046). However, this association did not remain statistically significant after false discovery rate adjustment (q = 0.276). Age, sex, subscapularis tear grade, supraspinatus tear type, and tear location relative to the LHBT were not significantly associated with MRI cable visibility (all *p* > 0.05). Factors associated with MRI cable visibility are summarized in Table 3. FDR-adjusted q values are provided in Supplementary Table S2.

### 3.6. Cable Visibility and Preoperative Range of Motion

No statistically significant differences in active abduction or active forward elevation were detected according to arthroscopic cable visibility in the overall cohort (*p* = 0.368 and *p* = 0.547, respectively). This finding persisted in subgroup analyses restricted to full-thickness tears not reaching the LHBT (abduction, *p* = 0.464; forward elevation, *p* = 0.698) and to full-thickness tears reaching or extending anterior to the LHBT (abduction, *p* = 0.286; forward elevation, *p* = 0.734). The relationship between cable visibility, cable dimensions, and preoperative range of motion is summarized in Table 4.

Similarly, no statistically significant differences in active abduction or forward elevation were detected according to tear location relative to the LHBT (*p* = 0.174 and *p* = 0.163, respectively). No statistically significant differences in these range-of-motion measures were detected according to MRI cable visibility (*p* = 0.748 and *p* = 0.837, respectively).

### 3.7. Preoperative Range of Motion

The mean active abduction was 94 ± 33° (range, 0–180°), and the mean active forward elevation was 105 ± 34° (range, 0–180°).

### 3.8. Factors Associated with MRI-Measured Cable Dimensions

No statistically significant associations were detected between cable width or cable thickness and age, sex, subscapularis tear grade, supraspinatus tear type, tear location, or arthroscopic cable visibility (all *p* > 0.05). No statistically significant correlations were detected between cable width or thickness and preoperative active abduction or forward elevation (all *p* > 0.05). Factors associated with MRI-measured cable width and thickness are summarized in Table 5.

### 3.9. Rotator Cable Visibility on Arthroscopy and MRI

**Figure 2:**
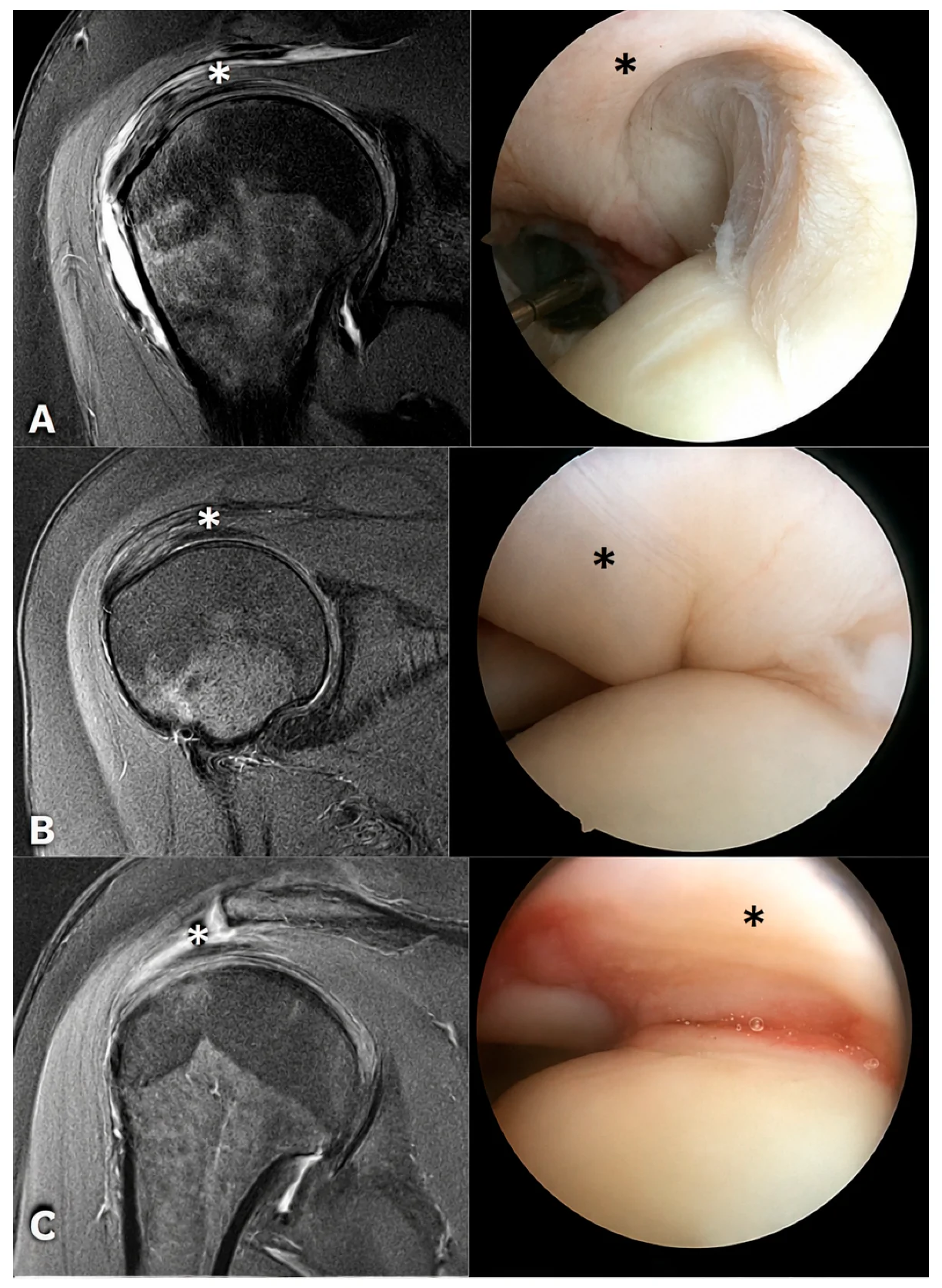
Representative MRI ((**left**), oblique coronal view) and corresponding arthroscopic (**right**) images from three different patients with right shoulders in whom the rotator cable was identifiable on both MRI and arthroscopy. (**A**–**C**) Representative MRI and corresponding arthroscopic images from three different patients. Each letter (A, B, and C) represents a different patient. * indicates the rotator cable.

**Figure 3:**
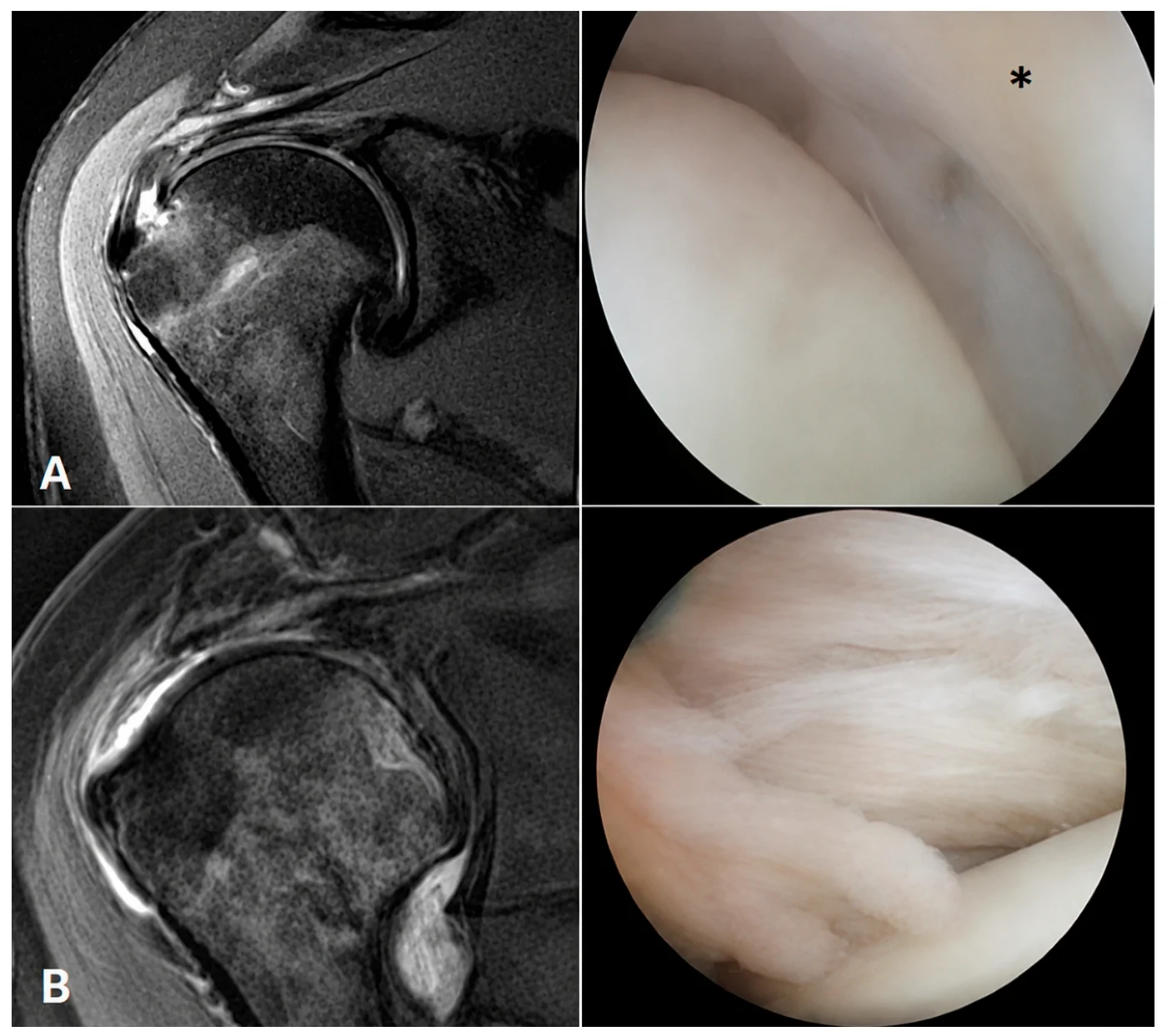
Representative arthroscopic (**right**) and MRI (**left**) images of the right shoulder. (**A**) The rotator cable was visible on arthroscopy but not identifiable on MRI. (**B**) The rotator cable was not visible on either arthroscopy or MRI. * indicates the rotator cable.

The rotator cable was visible on arthroscopy in 79 of 128 patients (61.7%) and was not visible in 49 patients (38.3%). Among the 58 patients with available preoperative MRI studies, the rotator cable was identified on MRI in 38 patients (65.5%) and was not identified in 20 patients (34.5%). Representative MRI and arthroscopic examples are shown in Figure 2 and Figure 3.

### 3.10. Agreement Between MRI and Arthroscopic Visualization

Among the 58 patients evaluated using both modalities, the rotator cable was visible on both arthroscopy and MRI in 26 patients and was not visible on either modality in 13 patients. In seven patients, the cable was visible on arthroscopy but not on MRI, whereas in 12 patients, it was visible on MRI but not on arthroscopy.

The positive percent agreement of MRI relative to arthroscopic visualization was 78.8% (95% CI, 61.1–91.0%), the negative percent agreement was 52.0% (95% CI, 31.3–72.2%), and the overall percent agreement was 67.2% (95% CI, 53.7–79.0%). Cohen’s kappa was 0.315 (95% CI, 0.090–0.540; *p* = 0.011), indicating fair agreement between the two modalities. Agreement measures are summarized in Table 6.

### 3.11. MRI Measurements of the Rotator Cable

Among the 38 patients in whom the rotator cable was identified on MRI, the mean cable width was 10.5 ± 3.5 mm (range, 3–16 mm), and the mean cable thickness was 0.90 ± 0.23 mm (range, 0.5–1.5 mm). The oblique coronal plane was the most useful plane for measuring both cable width and thickness. On oblique sagittal images, the cable appeared as a continuous hypointense longitudinal band along the articular margin of the supraspinatus and infraspinatus tendons, with anterior continuity toward the coracohumeral ligament.

## 4. Discussion

The present study evaluated arthroscopic and MRI visibility of the rotator cable in patients undergoing shoulder arthroscopy for supraspinatus tears and examined factors associated with cable detectability and its relationship with preoperative active range of motion. The rotator cable was identifiable in approximately two-thirds of patients on both arthroscopy and MRI, with fair agreement between the two modalities. In univariable analyses, arthroscopic cable visibility was associated with age and several tear-related factors; however, after multivariable adjustment, age was no longer independently associated with cable non-visibility. Higher Lafosse grade remained independently associated with cable non-visibility in the overall cohort, whereas increasing tear size remained independently associated with cable non-visibility among patients with full-thickness tears. Within the MRI subgroup, the unadjusted association between massive tear size and reduced cable visibility did not remain statistically significant after false discovery rate adjustment. No statistically significant associations were detected between cable visibility or MRI-measured cable dimensions and preoperative active abduction or forward elevation; however, these findings should not be interpreted as evidence of absence of a functional association.

### 4.1. Rotator Cable Visibility and Agreement Between Modalities

The arthroscopic cable visibility rate of 61.7% observed in the present study is within the broad range previously reported in arthroscopic series, which ranges from 10% to 100%. Similarly, the MRI visibility rate of 65.5% is consistent with previous non-contrast MRI studies reporting visualization rates between 62% and 91.7% [17,18]. Cadaveric studies have generally reported higher detection rates, approaching 100% in many series [6,18,19,20], which may reflect the greater spatial resolution and direct tissue access provided by dissection compared with imaging or arthroscopic visualization.

MRI and arthroscopy demonstrated fair agreement in the assessment of rotator cable visibility. Overall percent agreement was 67.2% (95% CI, 53.7–79.0%), with a Cohen’s kappa of 0.315 (95% CI, 0.090–0.540; *p* = 0.011). The positive percent agreement was 78.8% (95% CI, 61.1–91.0%), whereas the negative percent agreement was 52.0% (95% CI, 31.3–72.2%). Although positive percent agreement was relatively high, negative percent agreement was lower and imprecisely estimated, as reflected by its wide confidence interval. Discordant findings were observed in a substantial proportion of patients: in 7 patients, the cable was visible on arthroscopy but not on MRI, whereas in 12 patients, it was visible on MRI but not on arthroscopy.

This discordance should not be interpreted as evidence that MRI is diagnostically unreliable, because neither MRI nor arthroscopy provides a definitive assessment of structural cable integrity. Arthroscopic visualization was used only as an operational comparator. Rather, the observed differences may reflect modality-specific differences in tissue visualization, imaging plane, partial-volume effects, tissue degeneration, joint distension, and shoulder positioning. Future studies using optimized imaging protocols, such as ABER positioning or higher-resolution sequences, may help improve preoperative assessment of the rotator cable.

### 4.2. Clinical and Tear-Related Factors Influencing Arthroscopic Cable Visibility

Increasing age was associated with reduced arthroscopic cable visibility in the univariable analysis [12,21,22,23]. However, this association did not remain statistically significant after adjustment for sex, tear type, and Lafosse grade. This suggests that the apparent effect of age may be partly explained by the greater prevalence or severity of tear-related abnormalities in older patients. This finding may appear counterintuitive in light of Burkhart’s suspension bridge model, which suggests that the cable may become more prominent as the rotator crescent thins and load transfer shifts toward the cable [24,25]. However, in patients with symptomatic supraspinatus tears, advancing age may also be accompanied by progressive tendon degeneration, poorer tissue quality, and more complex tear patterns, which may reduce arthroscopic cable prominence or make the cable more difficult to distinguish intraoperatively.

Arthroscopic cable visibility was also reduced with increasing tear size. The cable was visible in most small and medium tears, was less frequently identified in large tears, and was not identified in any patient with a massive tear. In the multivariable analysis restricted to full-thickness tears, increasing tear size remained independently associated with cable non-visibility (adjusted OR per one-category increase, 2.78; 95% CI, 1.19–6.52; *p* = 0.019). Importantly, non-visualization of the cable in massive tears cannot be equated with complete cable rupture. Tendon retraction, advanced degenerative changes, distortion of the normal rotator cuff architecture, and loss of recognizable anatomical landmarks may make a residual or partially preserved cable difficult to identify arthroscopically. Because the present study assessed detectability rather than histological continuity or biomechanical competence, the data cannot distinguish true cable disruption from failure to visualize an anatomically altered or partially preserved cable.

The relationship between tear extension relative to the LHBT and cable visibility is clinically relevant. Tears extending anterior to the LHBT had substantially lower cable visibility than tears not reaching the LHBT or reaching only its posterior aspect. This supports the concept that anterior tear propagation may compromise the anterior cable region or its surrounding anatomic landmarks. The concurrent reduction in cable visibility with advancing subscapularis tear grade further suggests an anatomic relationship between the anterior cable region, the biceps pulley, and the subscapularis tendon. This interpretation is consistent with biomechanical data showing that isolated supraspinatus sectioning does not significantly alter glenohumeral translation, whereas combined anterior and posterior rotator cuff deficiency does [26,27]. Nevertheless, in the full-thickness multivariable model, tear extension relative to the LHBT and Lafosse grade were no longer statistically significant after adjustment, whereas tear size remained independently associated with cable non-visibility. These findings suggest that tear size may account for at least part of the unadjusted associations between cable visibility and other tear-related characteristics.

### 4.3. MRI Cable Visibility and Dimensions

Within the MRI subgroup, massive tears showed lower cable visibility than non-massive tears in the unadjusted analysis (25.0% vs. 67.9%; *p* = 0.046). However, this association did not remain statistically significant after false discovery rate adjustment (q = 0.276). Age, sex, subscapularis tear grade, supraspinatus tear type, and tear location relative to the LHBT were not statistically significantly associated with MRI visibility. Therefore, the massive-tear finding should be regarded as an exploratory unadjusted association rather than a robust independent finding.

The mean MRI-measured cable width was 10.5 ± 3.5 mm, and the mean thickness was 0.90 ± 0.23 mm. These values are generally comparable with previously reported imaging and anatomic data [2,4,18,28] although the measured thickness in the present study was lower than that reported in several cadaveric studies [4,14,19,20] (Table 7). This discrepancy may be explained by the spatial resolution limits of standard MRI, slice thickness, partial-volume effects, and differences in measurement methodology. No statistically significant associations were detected between MRI-measured cable width or thickness and demographic factors, tear characteristics, arthroscopic cable visibility, or preoperative active range of motion. Given the limited MRI sample size, these findings should be interpreted cautiously and do not exclude smaller or moderate associations.

An additional consideration is the multiplicity of the univariable comparisons performed in this study. In sensitivity analyses using false discovery rate adjustment, the principal arthroscopy-based associations remained robust, whereas the unadjusted MRI finding for massive tears did not remain statistically significant. This supports a more cautious interpretation of the MRI subgroup results, particularly given the limited sample size.

### 4.4. Functional Implications of Rotator Cable Visibility

Although no statistically significant association was detected between cable visibility and preoperative active abduction or forward elevation, this finding should not be interpreted as evidence that the rotator cable has no functional relevance [2,31]. No statistically significant differences in these range-of-motion measures were detected according to arthroscopic or MRI cable visibility, and no statistically significant correlations were detected between MRI-measured cable width or thickness and preoperative active range of motion.

Several explanations are possible. First, cable visibility may not correspond to structural or biomechanical integrity: a visible cable may be functionally compromised, whereas a poorly visualized cable may retain mechanical competence. Second, active shoulder motion is influenced by multiple factors that were not systematically available in this retrospective dataset, including pain severity, tear chronicity, muscle atrophy, fatty infiltration, pseudoparalysis, deltoid and scapulothoracic compensation, and patient effort. Active abduction and forward elevation therefore represent only limited components of shoulder function and may be insufficient to identify the functional contribution of the rotator cable. Third, the functional contribution of the rotator cable may become clinically apparent mainly in advanced tear patterns, particularly massive tears with substantial involvement of cable attachment regions. This interpretation is consistent with Denard et al., who reported an association between cable attachment disruption and pseudoparalysis in patients with massive rotator cuff tears [11]. Accordingly, the present negative findings should be regarded as inconclusive rather than as proof of absence of a functional association. Smaller associations or associations confined to specific clinical subgroups cannot be excluded.

From a surgical perspective, intraoperative identification of the rotator cable and its attachment regions may provide complementary anatomical information regarding tear morphology, the extent of rotator cuff disruption, and the remaining cuff anatomy and may potentially assist in planning repair configuration. However, the present study did not evaluate postoperative outcomes or whether cable-directed surgical decision-making improves clinical results. Therefore, cable visibility should not be considered a direct surrogate marker of shoulder function, and the clinical benefit of identifying the cable or altering surgical strategy according to its appearance remains to be established in prospective studies.

## 5. Limitations

Several limitations should be acknowledged. First, the retrospective design introduces potential selection bias. The MRI subgroup was not fully representative of the overall cohort, as partial-thickness tears were more frequent among patients included in the standardized MRI analysis. Although no statistically significant differences were detected in age, sex, arthroscopic cable visibility, range of motion, or full-thickness tear characteristics, selection bias related to MRI availability cannot be excluded. Therefore, the MRI-based findings should be interpreted cautiously and may not be fully generalizable to the entire study cohort.

Second, no a priori sample size calculation was performed because the sample size was determined by the number of eligible patients available during the retrospective study period. The unequal group sizes and the limited number of patients with standardized MRI review reduced statistical precision. Sensitivity analysis indicated that the MRI subgroup could detect only relatively large standardized between-group differences and correlations of approximately |r| = 0.44 or greater with 80% power. Therefore, smaller or moderate associations may have remained undetected, and nonsignificant MRI-based findings should not be interpreted as evidence of absence of an association.

Third, there was no control group of asymptomatic individuals or patients without rotator cuff tears, preventing comparison of cable visibility across different shoulder conditions. Fourth, MRI examinations were performed without ABER positioning, which may improve visualization of the rotator cable. Fifth, shoulder function was assessed only using preoperative active abduction and forward elevation. Pain severity, tear chronicity, pseudoparalysis, muscle atrophy, fatty infiltration, compensatory scapulothoracic motion, and validated patient-reported outcome measures were not systematically available. Therefore, residual confounding is likely, and the study may not have captured the full functional implications of rotator cable pathology.

Sixth, MRI and arthroscopic assessments were each performed by a single observer, and repeat blinded evaluations were not available. Consequently, intraobserver and interobserver reproducibility could not be assessed. Because cable visibility is partly subjective, observer-related misclassification and measurement variability cannot be excluded.

Finally, this study assessed whether the rotator cable could be identified using arthroscopy or MRI; it did not directly evaluate histological continuity, mechanical competence, or integrity of the cable attachment sites. Cable non-visibility may reflect structural disruption or degeneration, but it may also result from obscuration by tear morphology, poor tissue quality, limitations in imaging resolution or plane, joint positioning, or technical constraints during arthroscopic assessment. Therefore, non-visibility should not be interpreted as definitive evidence of anatomical absence or complete structural failure.

## 6. Conclusions

The rotator cable was visible on arthroscopy in 61.7% and on MRI in 65.5% of patients with supraspinatus tears, and standard MRI demonstrated fair agreement with arthroscopic visualization of the rotator cable. After multivariable adjustment, higher Lafosse grade was independently associated with cable non-visibility in the overall cohort, whereas increasing tear size was independently associated with cable non-visibility among patients with full-thickness tears. Age was not independently associated with cable non-visibility after adjustment for tear-related characteristics. MRI subgroup findings should be interpreted cautiously because the unadjusted association with massive tear size did not remain significant after adjustment for multiple comparisons. Within the limitations of the available functional measures, no statistically significant associations were detected between cable visibility or MRI-measured cable dimensions and preoperative active abduction or forward elevation. These findings do not exclude a functional role of the rotator cable or associations with functional outcomes not assessed in the present study. The observed discordance between MRI and arthroscopy likely reflects the inherent and modality-specific limitations of both assessments, and neither modality should be interpreted as a definitive indicator of structural cable integrity. From a surgical perspective, identification of the rotator cable may provide complementary anatomical information regarding the extent and configuration of rotator cuff disruption and the remaining cuff anatomy; however, because postoperative outcomes and cable-directed surgical decision-making were not evaluated, the clinical benefit of identifying the cable cannot be established from the present data. Prospective studies are needed to determine whether cable visualization should influence repair strategy or postoperative outcomes.

## Figures and Tables

**Table 1:** Factors affecting cable visibility on arthroscopy.

Variable	Finding	*p* Value	Significant?
Age	Older age → lower visibility (median 58 vs. 55 years)	0.005	Yes ✓
Subscapularis tear stage (Lafosse)	Higher stage → lower visibility	0.011	Yes ✓
Tear size (full-thickness)	Larger tear → lower visibility; massive tears 0%	<0.001	Yes ✓
Partial vs. full-thickness tear	Partial tears higher visibility (81.8% vs. 54.7%)	0.011	Yes ✓
Tear localisation (relative to LHBT)	Tears extending anterior to LHBT: only 25% visible	<0.001	Yes ✓
Sex	Male 67.4% vs. female 58.5%—no difference	0.424	No ✗

Arrows (→) indicate the direction of the observed association; ✓ indicates a statistically significant association (*p* < 0.05), whereas ✗ indicates no statistically significant association (*p* ≥ 0.05).

**Table 2:** Multivariable logistic regression analyses of factors associated with arthroscopic rotator cable non-visibility. (**A**) Overall cohort, *n* = 128. (**B**) Patients with full-thickness tears, *n* = 95.

(**A**)			
**Variable**	**Adjusted OR**	**95% CI**	***p* Value**
Age, per 10-year increase	1.42	0.94–2.16	0.098
Male sex vs. female sex	0.73	0.32–1.68	0.462
Full-thickness vs. partial-thickness tear	1.76	0.57–5.46	0.324
Lafosse grade, per one-grade increase	1.43	1.04–1.97	0.028
(**B**)			
**Variable**	**Adjusted OR**	**95% CI**	***p* Value**
Age, per 10-year increase	1.24	0.73–2.10	0.434
Male sex vs. female sex	0.59	0.20–1.74	0.343
Tear size, per one-category increase	2.78	1.19–6.52	0.019
Lafosse grade, per one-grade increase	1.26	0.85–1.86	0.256
Reaching posterior aspect of LHBT vs. not reaching LHBT	0.74	0.21–2.56	0.631
Extending anterior to LHBT vs. not reaching LHBT	2.68	0.67–10.66	0.162

The dependent outcome was arthroscopic cable non-visibility (0 = visible, 1 = not visible). Odds ratios greater than 1 therefore indicate increased odds of cable non-visibility. Age was entered per 10-year increase. Tear size was entered as an ordinal variable coded from 1 (small) to 4 (massive). Lafosse grade was entered as an ordinal variable coded from 0 (no subscapularis tear) to 5 (Lafosse Type V). All variables were entered simultaneously. OR, odds ratio; CI, confidence interval; LHBT, long head of the biceps tendon.

**Table 3:** Factors affecting cable visibility on MRI.

Variable	Finding	*p* Value	Significant?
Tear size (massive vs. non-massive)	Massive: 25% visible vs. 68% non-massive	0.046	Yes ✓
Age	No difference (median 54.5 vs. 57 years)	0.915	No ✗
Sex	Female 60.5% vs. male 39.5%—no difference	0.670	No ✗
Subscapularis tear stage	No difference across stages	0.651	No ✗
Tear localisation (LHBT)	No difference across groups	0.374	No ✗
Partial vs. full-thickness tear	No significant difference (76.2% vs. 59.5%)	0.317	No ✗

✓ indicates statistical significance (*p* < 0.05), whereas ✗ indicates no statistical significance (*p* ≥ 0.05).

**Table 4:** Effect on physical examination (range of motion).

Variable	Finding	*p* Value	Significant?
Arthroscopic cable visibility → Abduction/Flexion	No difference between groups	0.368/0.547	No ✗
MRI cable visibility → Abduction/Flexion	No significant relationship	0.748/0.837	No ✗
MRI cable width/thickness → ROM	No correlation (r ≈ 0.07–0.15)	>0.38	No ✗
Tear localisation → Abduction/Elevation	No difference across localisation groups	0.174/0.163	No ✗

→ indicates the direction of the assessed association; ✗ indicates no statistically significant association (*p* ≥ 0.05).

**Table 5:** Factors affecting MRI cable width and thickness.

Variable	Finding	*p* Value	Significant?
Age → width/thickness	No correlation	0.487/0.396	No ✗
Sex → width/thickness	No difference	0.182/0.516	No ✗
Subscapularis tear stage	No difference across groups	0.389/0.552	No ✗
Partial vs. full-thickness tear	No difference	0.787/0.438	No ✗
Tear localisation	No difference across groups	0.903/0.157	No ✗
Arthroscopic cable visibility	No effect on MRI measurements	0.370/0.740	No ✗

→ indicates the direction of the assessed association; ✗ indicates no statistically significant association (*p* ≥ 0.05).

**Table 6:** Agreement between MRI and arthroscopic visualization of the rotator cable. (**A**) Cross-classification of findings. (**B**) Agreement measures.

(**A**)			
**Arthroscopic Visualization**	**MRI Visible**	**MRI Not Visible**	**Total**
Visible	26	7	33
Not visible	12	13	25
Total	38	20	58
(**B**)			
**Measure**	**Estimate**	**95% CI**	
Positive percent agreement	78.8%	61.1–91.0%	
Negative percent agreement	52.0%	31.3–72.2%	
Overall percent agreement	67.2%	53.7–79.0%	
Cohen’s kappa	0.315	0.090–0.540	

Arthroscopic visualization was used as an operational comparator and not as a gold standard for structural cable integrity. Positive percent agreement represents the proportion of arthroscopically visible cables that were also visible on MRI. Negative percent agreement represents the proportion of cables not visible on arthroscopy that were also not visible on MRI. Exact binomial 95% confidence intervals are reported for percentage agreement measures. The *p* value for Cohen’s kappa was 0.011. MRI, magnetic resonance imaging; CI, confidence interval.

**Table 7:** Literature comparison: Rotator cable width and thickness.

Study (Year)	Width (mm)	Thickness (mm)	Method	Notes
Clark et al. (1990) [2]	~10	—	Cadaver	Described as ‘transverse band’
Burkhart et al. (1993) [1]	12.05	4.72	Cadaver	*n* = 20 shoulders
Kolts et al. (2000) [20]	~10	—	Cadaver	Ligamentum semicircular humeri
Morag et al. (2006) [29]	4.5	1.2	Cadaver + USG	Small sample
Sconfienza et al. (2012) [25]	5.8/4.0 *	1.5/1.1 *	USG	* Young vs. elderly asymptomatic
Macarini et al. (2011) [30]	—	2.8 ± 0.3	MRI (no contrast)	
Orlandi et al. (2012) [22]	5.6/4.2 *	1.3/1.2 *	USG	* Young vs. elderly
Morag et al. (2012) [21]	11.5	1.86	USG	Asymptomatic volunteers
Gyftopoulos et al. (2013) [9]	12.4 ± 3.1	1.9 ± 0.5	MRI (no contrast)	Intact cuff
Choo et al. (2014) [8]	8.55–14.22	1.07–1.99	Indirect MR arthrogram	4 groups by tear type
Podgorski et al. (2019) [23]	8.95 ± 1.54	1.56 ± 0.70	Cadaver	
Arai et al. (2020) [5]	6.18 ± 1.43	—	Cadaver	Macro + microscopic
Zink et al. (2021) [31]	10.9/7.7 ^†^	—	Cadaver	^†^ Bursal vs. articular side
Fondin et al. (2024) [13]	—	1.44 ± 0.20	Cadaver	Biomechanical study
Present study (2026)	10.5 ± 3.5	0.90 ± 0.23	MRI (no contrast)	*n* = 38; range 3–16/0.5–1.5 mm

MRI = Magnetic Resonance Imaging; USG = Ultrasonography.

## Data Availability

The data supporting the findings of this study are available from the corresponding author upon reasonable request. The data are not publicly available because of patient privacy and ethical restrictions.

## Supplementary Materials

The following supporting information can be downloaded at: https://www.mdpi.com/article/10.3390/jcm15176535/s1, Table S1: Comparison of patients included and not included in the standardized MRI analysis; Table S2: False discovery rate-adjusted q values for univariable analyses.

## References

[1] Burkhart S.S., Esch J.C., Jolson R.S. (1993). The rotator crescent and rotator cable: An anatomic description of the shoulder’s “suspension bridge”. Arthroscopy.

[2] Clark J., Sidles J.A., Matsen F.A. (1990). The relationship of the glenohumeral joint capsule to the rotator cuff. Clin. Orthop. Relat. Res..

[3] Burkhart S.S., Nottage W.M., Ogilvie-Harris D.J., Kohn H.S., Pachelli A. (1994). Partial repair of irreparable rotator cuff tears. Arthroscopy.

[4] Burkhart S.S. (1992). Fluoroscopic comparison of kinematic patterns in massive rotator cuff tears: A suspension bridge model. Clin. Orthop. Relat. Res..

[5] Arai R., Matsuda S. (2020). Macroscopic and microscopic anatomy of the rotator cable in the shoulder. J. Orthop. Sci..

[6] Kask K., Kolts I., Lubienski A., Russlies M., Leibecke T., Busch L.C. (2008). Magnetic resonance imaging and correlative gross anatomy of the ligamentum semicirculare humeri (rotator cable). Clin. Anat..

[7] Bureau N.J., Blain-Pare E., Tetreault P., Rouleau D.M., Hagemeister N. (2016). Sonographic visualization of the rotator cable in patients with symptomatic full-thickness rotator cuff tears: Correlation with tear size, muscular fatty infiltration and atrophy, and functional outcome. J. Ultrasound Med..

[8] Choo H.J., Lee S.J., Kim D.W., Park Y.M., Kim J.H. (2014). Assessment of the rotator cable in various rotator cuff conditions using indirect MR arthrography. Acta Radiol..

[9] Gyftopoulos S., Bencardino J., Nevsky G., Hall G., Soofi Y., Desai P., Jazrawi L., Recht M.P. (2013). Rotator cable: MRI study of its appearance in the intact rotator cuff with anatomic and histologic correlation. AJR Am. J. Roentgenol..

[10] Cho N.S., Moon S.C., Hong S.J., Bae S.H., Rhee Y.G. (2017). Comparison of clinical and radiological results in the arthroscopic repair of full-thickness rotator cuff tears with and without the anterior attachment of the rotator cable. Am. J. Sports Med..

[11] Denard P.J., Koo S.S., Murena L., Burkhart S.S. (2012). Pseudoparalysis: The importance of rotator cable integrity. Orthopedics.

[12] Morag Y., Jamadar D.A., Miller B., Brandon C., Gandikota G., Jacobson J.A. (2013). Morphology of large rotator cuff tears and of the rotator cable and long-term shoulder disability in conservatively treated elderly patients. J. Comput. Assist. Tomogr..

[13] Fondin M., Miroir M., Guillin R., Landreau J., Ghukasyan G., Fautrel A., Ropars M., Morandi X., Timoh K.N., Le Cam J.-B. (2024). Mechanical strength of the rotator cuff and cable interface: A complete histological and biomechanical study. Surg. Radiol. Anat..

[14] Wieser K., Rahm S., Farshad M., Ek E.T., Gerber C., Meyer D.C. (2013). Stitch positioning influences the suture hold in supraspinatus tendon repair. Knee Surg. Sports Traumatol. Arthrosc..

[15] DeOrio J.K., Cofield R.H. (1984). Results of a second attempt at surgical repair of a failed initial rotator-cuff repair. J. Bone Jt. Surg. Am..

[16] Lafosse L., Jost B., Reiland Y., Audebert S., Toussaint B., Gobezie R. (2007). Structural integrity and clinical outcomes after arthroscopic repair of isolated subscapularis tears. J. Bone Jt. Surg. Am..

[17] Davis D.E., Lee B., Aleem A., Abboud J., Ramsey M. (2020). Interobserver reliability of the rotator cable and its relationship to rotator cuff congruity. J. Shoulder Elb. Surg..

[18] Habermeyer P., Krieter C., Tang K.L., Lichtenberg S., Magosch P. (2008). A new arthroscopic classification of articular-sided supraspinatus footprint lesions: A prospective comparison with Snyder’s and Ellman’s classification. J. Shoulder Elb. Surg..

[19] Gyftopoulos S., Bencardino J.T., Immerman I., Zuckerman J.D. (2012). The rotator cable: Magnetic resonance evaluation and clinical correlation. Magn. Reson. Imaging Clin. N. Am..

[20] Kolts I., Busch L.C., Tomusk H., Arend A., Eller A., Merila M., Russlies M. (2000). Anatomy of the coracohumeral and coracoglenoidal ligaments. Ann. Anat..

[21] Morag Y., Jamadar D.A., Boon T.A., Bedi A., Caoili E.M., Jacobson J.A. (2012). Ultrasound of the rotator cable: Prevalence and morphology in asymptomatic shoulders. AJR Am. J. Roentgenol..

[22] Orlandi D., Sconfienza L.M., Fabbro E., Ferrero G., Martini C., Lacelli F., Serafini G., Silvestri E. (2012). Preliminary ultrasound evaluation of the rotator cable in asymptomatic volunteers. J. Ultrasound.

[23] Podgorski M.T., Olewnik L., Grzelak P., Polguj M., Topol M. (2019). Rotator cable in pathological shoulders: Comparison with normal anatomy in a cadaveric study. Anat. Sci. Int..

[24] Roache P.B. (2021). Anterior cable tears in arthroscopic rotator cuff repairs. Arthrosc. Sports Med. Rehabil..

[25] Sconfienza L.M., Orlandi D., Fabbro E., Ferrero G., Martini C., Savarino E., Silvestri E. (2012). Ultrasound assessment of the rotator cuff cable: Comparison between young and elderly asymptomatic volunteers and interobserver reproducibility. Ultrasound Med. Biol..

[26] Sheah K., Bredella M.A., Warner J.J.P., Halpern E.F., Palmer W.E. (2009). Transverse thickening along the articular surface of the rotator cuff consistent with the rotator cable: Identification with MR arthrography and relevance in rotator cuff evaluation. AJR Am. J. Roentgenol..

[27] Su W.R., Budoff J.E., Luo Z.P. (2009). The effect of anterosuperior rotator cuff tears on glenohumeral translation. Arthroscopy.

[28] Wang L., Kang Y., Xie G., Cai J., Chen C., Yan X., Jiang J., Zhao J. (2021). Incomplete rotator cable did not cause rotator cuff dysfunction in case of rotator cuff tear: A biomechanical study. Arthroscopy.

[29] Morag Y., Jacobson J.A., Lucas D., Miller B., Brigido M.K., Jamadar D.A. (2006). US appearance of the rotator cable with histologic correlation: Preliminary results. Radiology.

[30] Macarini L., Muscarella S., Lelario M., Stoppino L., Scalzo G., Scelzi A., Armillotta M., Sforza N., Vinci R. (2011). Rotator cable at MR imaging: Considerations on morphological aspects and biomechanical role. Radiol. Med..

[31] Zink T.R., Schmidt C.C., Papadopoulos D.V., Blake R.J., Smolinski M.P., Davidson A.J., Spicer C.S., Miller M.C., Smolinski P.J. (2021). Locating the rotator cable during subacromial arthroscopy: Bursal- and articular-sided anatomy. J. Shoulder Elb. Surg..

