# Supplementary material for: Arthroscopic and MRI Visibility of the Rotator Cable in Supraspinatus Tears: Agreement, Associated Factors, and Relationship with Preoperative Range of Motion
Source: J Clin Med. 2026 Aug 24;15(17):6535. doi: 10.3390/jcm15176535 (PMC13566086; doi:10.3390/jcm15176535)
Supplement: Supplementary file 1 [file jcm-15-06535-s001.zip › jcm-4473802-supplementary.pdf]

Comparison of patients included and not included in the MRI analysis

Of the 128 patients, 58 were included in the standardized MRI analysis and 70 were not included. No statistically significant differences were detected between the groups in age, sex, arthroscopic cable visibility, active abduction, or active forward elevation (all $p > 0.05$). Partial-thickness tears were more frequent in the MRI subgroup than in the group without standardized MRI review (36.2% vs. 17.1%; $p = 0.014$). Among patients with full-thickness tears, neither the distribution of tear size nor tear extension relative to the LHBT differed significantly between the groups ($p > 0.05$). The comparison is presented in Supplementary Table S1.

Supplementary Table S1

**Characteristic**	**MRI analysis, n=58**	**No MRI analysis, n=70**	**p value**
Age, years, mean ± SD	56.2 ± 10.2	57.9 ± 9.9	0.320
Male sex, n (%)	21 (36.2)	25 (35.7)	0.954
Partial-thickness tear, n (%)	21 (36.2)	12 (17.1)	**0.014**
Arthroscopic cable visible, n (%)	33 (56.9)	46 (65.7)	0.307
Active abduction, degrees, median [IQR]	90 [90–110]	90 [70–100]	0.105
Active forward elevation, degrees, median [IQR]	105 [90–130]	100 [90–120]	0.390
**Full-thickness tears only**	**n=37**	**n=58**	
Tear size, n (%)			0.247
— Small	2 (5.4)	9 (15.5)	
— Medium	13 (35.1)	23 (39.7)	
— Large	14 (37.8)	20 (34.5)	
— Massive	8 (21.6)	6 (10.3)	
Tear extension relative to LHBT, n (%)			0.561
— Did not reach the LHBT	9 (24.3)	19 (32.8)	
— Reached the posterior aspect of the LHBT	14 (37.8)	17 (29.3)	
— Extended anterior to the LHBT	14 (37.8)	22 (37.9)	

Table footnote:

Values are presented as mean ± standard deviation, median [interquartile range], or number (percentage), as appropriate. P values were obtained using the independent-samples t test, Mann–Whitney U test, Pearson chi-square test, or Monte Carlo exact test, as appropriate. The Monte Carlo exact test was used for tear extension relative to the LHBT.

Supplementary Table S2. False discovery rate-adjusted q values for univariable analyses

A. Arthroscopic cable visibility

Variable	Unadjusted p	FDR-adjusted q
Age	0.005	0.010
Sex	0.424	0.424
Lafosse grade	0.011	0.013
Tear size	<0.001	<0.003
Tear type	0.011	0.013
Tear localization relative to LHBT	<0.001	<0.003

B. MRI cable visibility

Variable	Unadjusted p	FDR-adjusted q
Age	0.915	0.915
Sex	0.670	0.804
Lafosse grade	0.651	0.804
Tear localization relative to LHBT	0.374	0.748
Tear type	0.317	0.748
Massive vs. non-massive tear	0.046	0.276
